# Book Reviews

**Published:** 1912-08

**Authors:** 


### BOOK REVIEWS

---

DIAGNOSTIC METHODS, CHEMICAL, BACTERIOLOGICAL AND MICROSCOPICAL, *by Ralph W. Webster, M. D., Ph. D.*, Chicago; published by P. Blakiston's Son & Co., Philadelphia. 682 pages, 37 full page plates, 164 other illustrations, $4.50 (second revised edition.)

Both author and publisher have done their work well, making no attempt to secure a superficial appearance of novelty and originality by departing from established classifications and methods. The work, therefore enters directly into competition with others along the same line and we can scarcely express a preference.

---

TEXT BOOK FOR NURSES Anatomy, Physiology, Surgery and Medicine *by E. W. Hey Groves, M. S., F. R. C. S., and J. M. Fortescue-Brickdale, M. A., M. D.*, Bristol, Eng. Oxford University Press, London. 405 pages, 205 illustrations, including colored plates. Price $4.50.

Few books are so elegant typographically and pictorially. The volume of the work is significant when one considers that it is not primarily nor systematically one on nursing and that it does not contain dose tables and a discussion of materia medica as such. The authors have presented a resume, necessarily brief in details but astonishingly full in scope, of practically the whole science and art of medicine, with the endeavor to give the nurse a broad view of her duties or, rather, of the duties of the surgeon or physician whom she asists. There is a well known and well marked difference of opinion as to the advisability of attempting to ground the nurse in general principles of medicine, many holding that the appallingly self-sufficient nurse of English fiction results in real life, from too broad and too shallow instruction. Those who hold this view, will certainly censure the authors for preparing such a book though they must admit that it is an excellent one for the physician himself to use in refreshing his memory. Those holding the opposite view will find their ideal text book to place in the hands of the nurse.

---

THE CARE OF THE SKIN AND HAIR, *by William Allen Pusey, A. M., M. D.*, Prof. of Dermatology, University of Illinois; published by D. Appleton & Co., New York. 182 pages.

This practical little hand-book, by our class-mate at the University of Pennsylvania, is dedicated to his wife "without whose patience and indulgence such excursions as this were impossible." Translated in the light of our own experience, this means that physicians' wives make very practical and insistent demands for definite means of relief of conditions that we are too prone to regard as trical or that we attempt to pass off as inevitable or that we try to dodge as "out of our line." And yet, a great many women lose much that is best in life, just because, as a profession, we fail to "deliver the goods"—to descend to a vulgar expression, and we fail simply because we regard "the goods" as unimportant. The great majority of the "goods" that we order throughout life, are trival but we want them, never-the-less. Too often, the medical profession assumes the dignity of a store that will sell trousers but not neck-ties; of the grocer that will sell bread but not olives, forgetting that it is the little things of life that make for comfort. We trust that the wives of a good many other medical authors will make possible such excursions in various fields which the profession has plowed and planted but has not adequately fertilized, watered and kept free from weeds.

---

STOMATOLOGY IN GENERAL PRACTICE.—*Dr. H. P. Pickerill*, University of Otago, published by the Oxford University Press, 267 pages. Price $5.50.

While this work is on the border-line of dentistry, it has much of interest for the medical practitioner, apart from the inclusion of prophylactic methods. It deals with various conditions of the mouth, bones, etc., in addition to the teeth. We venture to disagree somewhat with the advice against the routine use of thread to remove debris from between the teeth. The discussion of salivary chemistry, though brief, is unusually good.

---

AUGUSTUS CHARLES BERNAYS, A Memoir, *by Thekla Bernays.* Published by the C. V. Mosby Co., St. Louis, 306 pages with portrait frontispiece, $2.00.

This is a very personal biography, by the distinguished surgeon's sister. If any criticism is possible it is that the biographer naturally could not deal technically with the professional and scientific side of his life and that, in her endeavor to present a frank picture of his life and nature, she rather emphasizes the faults for which he was loved, and the recreations—by no means reprehensible—which gave him strength for his real life work. Considered merely as a story, without regard to the interest in Dr. Bernays' accomplishments, it is fascinating reading.

---

Essays on Genito-Urinary Subjects, *by Dr. J. Bayard Clark* of New York, Wm. Wood & Co., New York, 174 pages, $1.25.

Several of the chapters are reprinted from periodicals. The tables showing the germicidal effects of various urethral and other antiseptics may be selected as the especially valuable part of the book. No pretense is made at covering the subject of genito-urinary disease in a systematic way, the chapters are hardly didactic but contain many useful side lights and are written in a most entertaining literary style.

---

Salvarsan in Syphilis and Allied Diseases (Supplement to A System of Syphilis), *J. E. R. McDonagh, F. R. C. S.*, London, The Oxford University Press, 152 pages, $3.00.

This epitomizes the literature on Salvarsan, to date, though by no means in the style of a review. The chemistry, practical value, relative safety, methods of administration, relations to the Wassermann reaction and other important points are thoroughly covered.

---

Infant Mortality and Milk Stations.—Special Report of the New York Milk Committee, 1912. 176 pages, paper covers, illustrated, $1.00.

While this report is edited by Dr. Philip Van Ingen and Mr. Paul Emmons Taylor, due credit should be given to a number of workers too numerous to mention, as well as the philanthropists behind the movement. A chart which, at a glance, resembles a genealogic record, indicates the organization of the charity. While the report covers similar work in the ten largest cities of the U. S., thus just admitting Buffalo, it deals mainly with the work in the Metropolis. Probably there is no other city in the civilized world presenting, on the one hand, a problem of infant mortality so terrible, and, on the other hand, so well adapted by concentration of population and its alien nature, to the work of relief which, in turn, can command more capital and labor, than in any other center. It would, however, be more accurate to say that there is no other city in the civilized world in which the problem and the class willing and able to attack it are more sharply separated.

